# The Neuronal EGF-Related Gene Nell2 Interacts with Macf1 and Supports Survival of Retinal Ganglion Cells after Optic Nerve Injury

**DOI:** 10.1371/journal.pone.0034810

**Published:** 2012-04-04

**Authors:** Yasunari Munemasa, Chang-Sheng Chang, Jacky M. K. Kwong, Haksu Kyung, Yasushi Kitaoka, Joseph Caprioli, Natik Piri

**Affiliations:** 1 Jules Stein Eye Institute, University of California Los Angeles, Los Angeles, California, United States of America; 2 Brain Research Institute, University of California Los Angeles, Los Angeles, California, United States of America; 3 Department Ophthalmology, St Marianna University School of Medicine, Kawasaki, Japan; University of Rochester, United States of America

## Abstract

Nell2 is a neuron-specific protein containing six epidermal growth factor-like domains. We have identified Nell2 as a retinal ganglion cell (RGC)-expressed gene by comparing mRNA profiles of control and RGC-deficient rat retinas. The aim of this study was to analyze Nell2 expression in wild-type and optic nerve axotomized retinas and evaluate its potential role in RGCs. Nell2-positive *in situ* and immunohistochemical signals were localized to irregularly shaped cells in the ganglion cell layer (GCL) and colocalized with retrogradely-labeled RGCs. No Nell2-positive cells were detected in 2 weeks optic nerve transected (ONT) retinas characterized with approximately 90% RGC loss. RT-PCR analysis showed a dramatic decrease in the Nell2 mRNA level after ONT compared to the controls. Immunoblot analysis of the Nell2 expression in the retina revealed the presence of two proteins with approximate MW of 140 and 90 kDa representing glycosylated and non-glycosylated Nell2, respectively. Both products were almost undetectable in retinal protein extracts two weeks after ONT. Proteome analysis of Nell2-interacting proteins carried out with MALDI-TOF MS (MS) identified microtubule-actin crosslinking factor 1 (Macf1), known to be critical in CNS development. Strong Macf1 expression was observed in the inner plexiform layer and GCL where it was colocalizied with Thy-1 staining. Since Nell2 has been reported to increase neuronal survival of the hippocampus and cerebral cortex, we evaluated the effect of Nell2 overexpression on RGC survival. RGCs in the nasal retina were consistently more efficiently transfected than in other areas (49% vs. 13%; n = 5, p<0.05). In non-transfected or pEGFP-transfected ONT retinas, the loss of RGCs was approximately 90% compared to the untreated control. In the nasal region, Nell2 transfection led to the preservation of approximately 58% more cells damaged by axotomy compared to non-transfected (n = 5, p<0.01) or pEGFP-transfected controls (n = 5, p<0.01).

## Introduction

Nell2 is a thrombospondin-1 (Tsp-1)-like glycoprotein containing six epidermal growth factor (EGF)-like and several von Willebrand factor C domains. The *nel* gene was originally isolated from a chick embryo-derived cDNA library [1]. It was ubiquitously expressed during development, but after hatching, the nel expression was restricted to neural tissues. In developing chicken retinotectal system, the expression of nel was localized in specific laminae of the tectum that retinal axons normally do not enter, suggesting that nel acts as an inhibitory guidance cue for retinal axons [2]. Two *nel*-like genes, *Nell1* and *Nell2*, were isolated from a human and rat brain cDNA library [3], [4]. The *Nell2* gene is more closely related to the *nel* gene than Nell1 and is expressed predominantly in the brain with the highest level in the hippocampus [3]. Similar to Tsp-1-induced signal transduction, Nell2 signaling has been shown to be mediated by extracellular signal-regulated kinase (Erk) and c-Jun N-terminal kinase (Jnk). Through the activation and suppression of Jnk and Erk, respectively, Nell2 has been reported to support the survival of neurons from the hippocampus and cerebral cortex [5]. Furthermore, since Erk and Jnk are implicated in the induction and inhibition of hippocampal long-term potentiation (LTP), it was suggested that Nell2 may play a role in this process [6], [7]. Evaluation of Nell2-deficient mice showed that this gene is an essential negative regulator of the neuronal activity important for LTP induction in the hippocampus [8].

We identified Nell2 as one of the genes, expression of which in the retina is restricted to retinal ganglion cell (RGC) during our recent analysis of RGC transcriptome [9]. RGCs provide the final neuronal output of the retina. They collect visual signals from bipolar and amacrine cells and transmit this information to the brain. Based on their morphological characteristics, such as soma size, dendritic field size, and dendritic ramification, at least 18 different types of RGCs have been identified in the human retina. Physiologically, these cells can be divided into several major types: 1) motion-sensitive parasol or magnocellular (M) RGCs; 2) color-sensitive midget or parvocellular (P) RGCs that are responsible for central visual acuity; 3) color opponent blue-yellow bistratified RGCs; 4) RGCs responsible for pupillary reaction; and 5) melanopsin-containing photosensitive RGCs responsible for the regulation of circadian rhythm [10], [11], [12], [13], [14], [15]. Degeneration of RGCs and their axons in the optic nerve leads to vision loss in various optic neuropathies including its most common form, glaucoma, which affects more than 70 million people worldwide and if left untreated, can lead to severe visual impairment and blindness (10% of total blindness cases in the U.S.)

Understanding the function of Nell2 in RGCs is of particular interest to us since it has been implicated in promoting survival, proliferation and differentiation of neuronal cells [5], [16], [17], [18]. As the pathophysiological mechanisms leading to RGC degeneration in glaucoma are not well understood and the current therapies are limited to the reduction of intraocular pressure, modulation of Nell2 expression could have a potential neuroprotective effect to stimulate RGC survival in this disease. In the present study we characterized Nell2 expression at the mRNA and protein level in control and optic nerve axotomized retinas, identified Nell2-interacting proteins in the retina and evaluated its cell protective role in ONT-induced RGC degeneration.

## Results

### Nell2 mRNA localization in the retina

**Figure 1:**
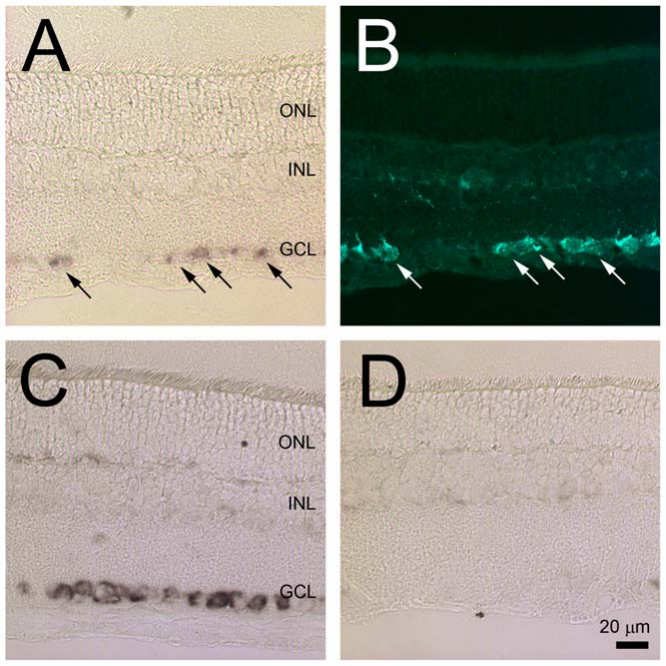
Nell2 mRNA expression in control and ONT retinas. A and C. Nell2 mRNA expression was localized to the cells in the GCL. D. No Nell2 positive cells were detected in retinal sections two weeks after ONT that leads to RGC degeneration. A and B. All Nell2 *in situ* hybridization signals were colocalized with RGCs retrogradely labeled with FG.

*In situ* hybridization (ISH) was the first experiment to confirm the results of the microarray data indicating that Nell2 is expressed in the retina predominantly or exclusively by RGCs. Using Nell2-specific antisense riboprobes, the expression of this gene in the adult rat retina was localized to the cells in the GCL (Fig. 1A). No Nell2 positive staining was detected in any other retinal layer. *Nell2* spatial expression was also analyzed in the retinas after ONT, which leads to rapid and specific degeneration of RGCs. The estimated number of surviving RGCs in the axotomy model used in this study was approximately 5–10%, which is consistent with the results of other studies showing up to 100% of RGC loss by two weeks after ONT [19]. In retinal sections analyzed by ISH two weeks after ONT, no Nell2 positive cells were detected. These results suggest that Nell2-positive cells in the GCL are RGCs. However, the GCL in the rodent retina contains both RGCs and non-RGCs, mostly displaced amacrine cells, in an approximately similar ratio. Therefore, to identity Nell2 positive cells, ISH was performed on retinas with retrogradely-labeled RGCs. Results presented in Fig. 1B show that Nell2-positive signals were colocalized with the Fluorogold (FG) retrogradely-labeled irregularly shaped large and medium size RGCs. Furthermore, consistent with RGC distribution in the rodent retina, Nell2-positive cells were present more densely in the posterior region compared to the peripheral retina. No positive reaction was present in the retinas subjected to ISH with the sense riboprobe used in these experiments as a negative control.

### Immunohistochemical localization of Nell2 protein in the retina

**Figure 2:**
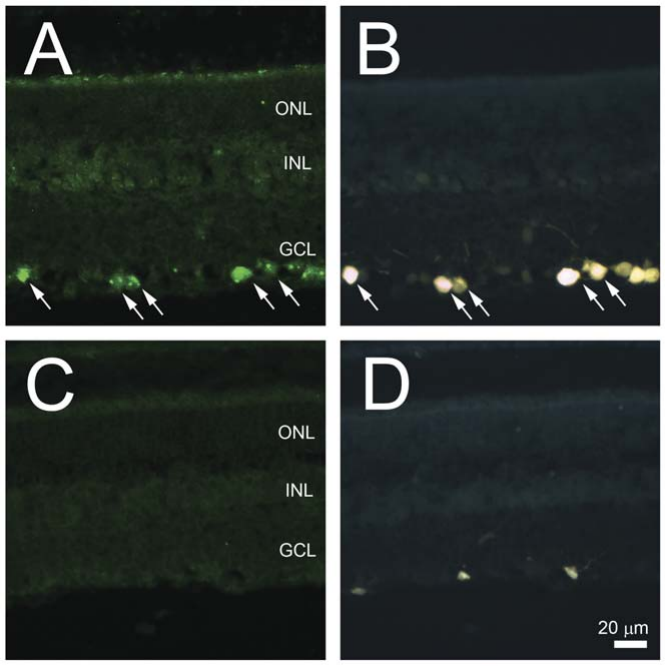
Immunohistochemical localization of Nell2 in control and ONT retinas. A and B. Co-localization of Nell2 expression with FG-labeled RGCs. The distribution of Nell2 protein in the retina was similar to its mRNA expression. The most abundant expression of these proteins was observed in the GCL. Furthermore, all Nell2-expressing cells in the GCL were colocalized with retrogradely-labeled RGCs. C and D. Loss of Nell2 immunohistochemical staining correlates with the loss of RGCs 2 weeks after ONT. Analysis of Nell2 protein expression 2 weeks after ONT showed no Nell2-positive cells in the retina. Very few FG-positive microglial cells are present.

To determine Nell2 spatial expression immunohistochemistry (IHC) was performed on untreated and axotomized rat retinal sections. The distribution of Nell2 protein in the retina was similar to its mRNA expression. The most abundant expression of these proteins was observed in the GCL (Fig. 2 A). Furthermore, all Nell2-expressing cells in the GCL were colocalized with retrogradely-labeled RGCs. Analysis of Nell2 protein expression 2 weeks after ONT showed no Nell2-positive cells in the retina (Fig. 2B). Very few small ramifying FG-stained microglial cell were present, however these cells were negative for Nell2 expression.

**Figure 3:**
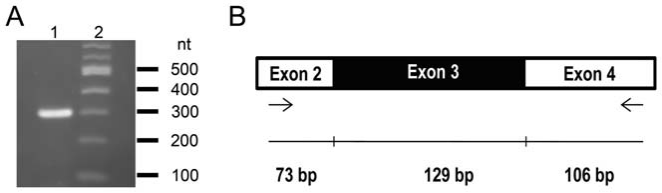
Expression of Nell2 splicing variants in the retina. A. RT-PCR analysis was performed to identify Nell2 isoforms. Only one ∼300 bp product corresponding to secreted Nell 2 isoform was detected (lane 1). Lane 2 is a 100 bp DNA ladder. B. Locations of the primers in the gene used for the PCR are indicated by arrows. The expected sizes of the products corresponding to secreted and non-secreted Nell2 isoforms are 308 bp and 179 bp, respectively.

A number of studies analyzing the Nell2 expression pattern have strongly suggested that this is a secreted glycoprotein that functions as a trophic factor as well as a signaling molecule during cell growth and differentiation [5], [18]. Later, a non-secreted isoform of Nell2 was found to be present throughout the cytoplasm and was suggested to be involved in intracellular signal pathways as an interacting partner of PKCβ1 [20]. Non-secreted Nell2 is generated by alternative splicing and lacks the third exon containing the terminal portion of the secretion signal peptide. The granular appearance of Nell2 immunohistochemical staining in RGCs (Fig. 2A) suggests its predominant localization in the intracellular compartments. This is in agreement with earlier observations of subcellular localization of Nell2 to the organelles associated with secretory processes, such as the Golgi apparatus and endoplasmatic reticulum. To identify Nell2 splicing isoforms expressed in the retina, an RT-PCR with primers flanking exon 3 was performed (Fig. 3A). In this experiment, the expected length of RT-PCR products are 308 bp and 179 bp that correspond to secreted and non-secreted variants of Nell2, respectively (Fig. 3B). Only one product of approximately 300 bp was detected, indicating the expression of secreted Nell2 isoform in the retina.

### RT-PCR and immunoblot (IB) analyses of Nell2 expression in the retina

**Figure 4:**
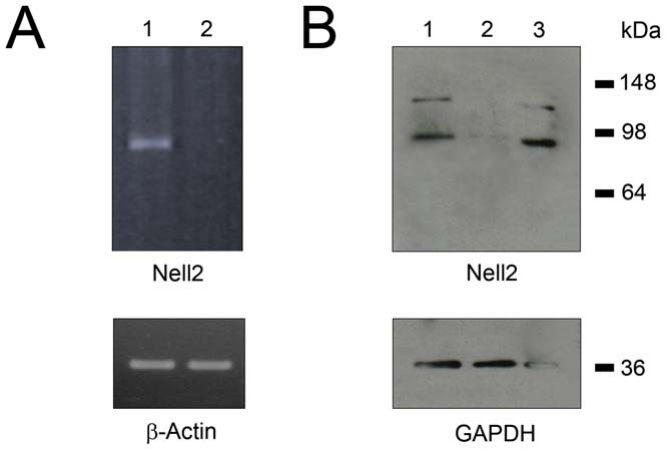
RT-PCR and immunoblot analyses of Nell2 expression in control and ONT retinas. A dramatic decrease in Nell2 mRNA (A) and protein (B) levels associated with the loss of RGCs was observed. B. Nell2 is represented in the retinal extract by 90 kDa and 140 kDa proteins, which are proposed to be non-glycosylated and glycosylated Nell2, respectively. Lanes: 1 – control retina; 2 – ONT retina; 3 – cortex.

RT-PCR of Nell2 showed a dramatic decrease in the level of Nell2 mRNA expression 2 weeks after ONT (Fig. 4A). This observation is in agreement with ISH data indicating a loss of Nell2-positive cells after axotomy. A western blot analysis of Nell2 protein in untreated retinas revealed the presence of two products with approximate molecular weight (MW) of 90 kDa and 140 kDa (Fig. 4B). A similar pattern was observed in a sample containing protein extract from cortex used in this experiment as a positive control. The intensity of the band corresponding to 90 kDa product was approximately 2 fold higher compared to 140 kDa product. Since the deduced MW of Nell2 is 91,402 Da, it is suggested that the ∼90 kDa and 140 kDa bands represent non-glycosylated and glycosylated Nell2, respectively. In retinas subjected to ONT, no 140 kDa product was present, whereas a very faint 90 kDa band was hardly detectable. This dramatic decrease in Nell2 protein level correlates with immunohistochemical and *in situ* data indicating the loss of Nell2-positive cells due to ONT-induced degeneration of RGCs.

### Nell2 interacts with microtubule-actin crosslinking factor 1 (Macf1) in RGCs

**Figure 5:**
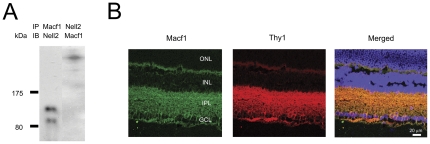
A reverse immunoprecipitation with a MACF1 antibody followed by immunoblot analysis with Nell2 antibodies and spatial expression of Macf1 in the retina. A. Two products with MW of approximately 90 and 140 kDa were detected by IB with Nell2 antibody following IP with anti Macf1. IB with MACF1 antibody of the lysate immunoprecipitated with Nell2 antibody showed a single high MW band corresponding to Macf1. B. Macf1 immunohistochemistry in retinal sections showed predominant Macf1 expression in the GCL and inner plexiform layerI (IPL). Expression of Macf1 in RGCs and their dendrites was shown by colocalization of Macf1 positive signals with Thy-1 staining, a commonly used marker for RGCs.

To gain insights into potential function of Nell2 in RGCs, Nell2 interacting proteins in the retina were identified with matrix-assisted laser desorption/ionisation-time of flight mass spectrometry (MALDI-TOF MS). A predominant high MW protein pulled down by the Nell2 antibody was detected in three consecutive experiments by coomassie blue staining. This anti-Nell2-immunoprecipitated product was subjected to MS analysis. Peptide mass fingerprinting and Mascot analysis of the amino acid sequence obtained from MALDI-TOF-TOF and MS-MS led to identification of this protein as Macf1 (product accession number gi 119627687 and MW ∼620 kDa). To further confirm the result obtained with MS, a reverse immunoprecipitation (IP) with an Macf1 antibody was performed. Two bands with MW of approximately 90 and 140 kDa were detected by IB with Nell2 antibody following IP with anti-Macf1 (Fig. 5A). IB with Macf1 antibody of the lysate immunoprecipitated with Nell2 antibody showed a single high MW band corresponding to Macf1 (Fig. 5A). The expression pattern of Macf1 in the retina was determined by immunohistochemistry. Strong Macf1 expression in the GCL and inner plexiform layer (IPL) was observed (Fig. 5B). Macf1 expression in RGCs was demonstrated by colocalization of Macf1 positive cells with Thy-1 staining, a commonly used marker for RGCs. The results of MALDI-TOF MS that were further corroborated with IP/IB and immunohistochemistry data provide strong evidence for the Nell2 interaction with Macf1 in RGCs.

### Overexpression of Nell2 in the retina and RGC survival

**Figure 6:**
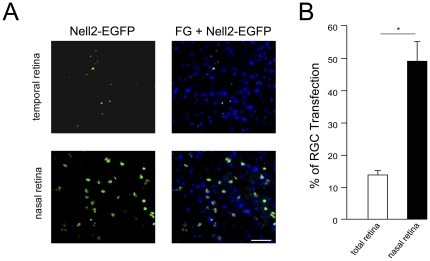
Electroporation-mediated *in vivo* transfection of RGCs with Nell2 expression construct. A. Co-localization of pNell2-EGFP-transfected cells with FG-labeled RGCs. A few EGFP-positive cells were present in temporal retinas. Most transfected cells were in the nasal retina. Most transfected cells in the retina were colocalized with RGCs. No EGFP staining was observed in non-transfected retinas. B. Quantitative analysis of ELP-mediated transfection efficiency. Approximately 49% of RGCs in the upper nasal region of the retina were transfected with pNell2-EGFP, whereas the average number of transfected RGCs across the entire retina was approximately 13% (n = 5). *p<0.05.

The notion that Nell2 could support the survival of injured RGCs was based on an earlier study [5] demonstrating a cell protective role of Nell2 in primary neuron cultures of the hippocampus and cerebral cortex. Nell2 fused to an EGFP expression construct was used to evaluate the *in vivo* effect of this protein on the survival of axotomized RGCs in an ONT rat model. Constructs were delivered to the retina by electroporation (ELP). First, we determined the efficiency of ELP-mediated RGC transfection by comparing the number of EGFP-positive cells colocalized with FG-labeled RGCs and the total number of FG-labeled RGCs (Fig. 6A). RGCs in the upper nasal retina were consistently more efficiently transfected than in other areas. Approximately 49% of RGC in the upper nasal area were transfected, whereas the transfection efficiency for the entire retina was only ∼13% (n = 5, p<0.05; Fig. 6B).

**Figure 7:**
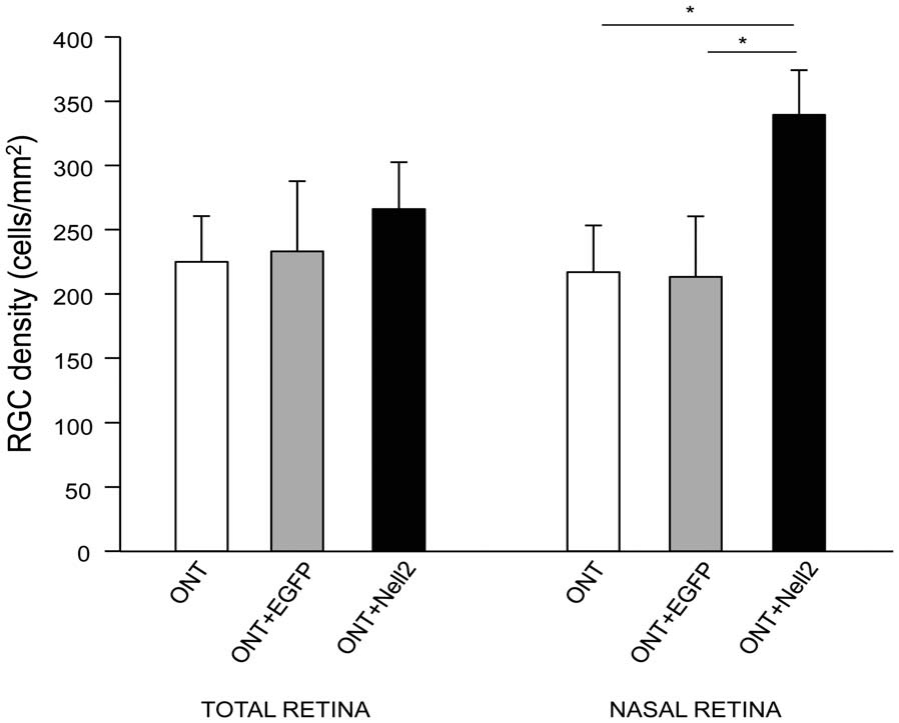
The neuroprotective effect of Nell2 expression on axotomized RGCs. The cell protective effect of Nell2 was correlated with the transfection efficiency. Nell2-mediated cell protective effect for the entire retina (approximately 13% of transfected RGCs) was calculated to be only about 10%. In the nasal region of the retina with highest numbers of transfected RGCs (∼49%), the Nell2 overexpession supported survival of approximately 58% more RGCs than in non-transfected (n = 5) or pEGFP-transfected (n = 5) control retinas. *p<0.01.

To evaluate the cell protective effect of Nell2 overexpression, the transfection was performed one day before ONT and RGCs were counted 2 weeks after ONT. Non-transfected pEGFP-transfected ONT retinas were used as controls. In non-transfected ONT retinas, the loss of RGCs was approximately 90% (218±36 cells/mm^2^ survived) compared to the untreated retinas (Fig. 7). No significant difference was observed in ONT-induced RGC loss in pEGFP-transfected versus non-transfected eyes. This was true for both the entire retina and the upper nasal region. The numbers of survived RGCs in pNell2-EGFP transfected retinas were approximately 265±38 and 344±31 per mm^2^ in the whole retina and upper nasal region, respectively (Fig. 6). Virtually all of the surviving RGCs in the nasal region were pNell2-EGFP-transfected. These results indicate that relatively efficient transfection of RGCs with a Nell2 expression construct (∼49% in this experiment) led to the preservation of approximately 58% more cells damaged by axotomy compared to the non-transfected (n = 5, p<0.01) or pEGFP-transfected controls (n = 5, p<0.01).

## Discussion

Nell2 has been identified as a gene, the expression of which in the retina is restricted or predominant in RGCs [9]. The present study was initiated with the aim to characterize Nell2 expression in the retina and evaluate its potential neuroprotective function. To our knowledge, this is the first study describing expression and potential role of Nell2 in the retina. Expression of this gene was analyzed at both mRNA and protein levels in wild type retinas and in retinas of the ONT model of RGC degeneration. ISH and immunohistochemistry were performed to determine the spatial expression of Nell2 in the retina. Nell2 mRNA was specifically localized to retrogradely-labeled RGCs confirming the results of the microarray analysis. Furthermore, both RT-PCR and ISH on retinal sections from ONT animals, in which ∼90% of RGCs degenerate by two weeks after the injury showed near complete loss of Nell2 mRNA expression. In agreement with the mRNA data, Nell2 protein expression was also localized to RGCs. A granular appearance of Nell2 immunoreactivity in RGC somas suggests an enriched localization of this protein in the intracellular compartments. This observation is in agreement with earlier reports on preferential localization of Nell2 at distinct subcellular structures, presumably at the endoplasmic reticulum, Golgi apparatus and vesicles [20]. Although, as noted above, both, secreted and cytosolic splicing isoforms have been identified for the Nell2 [20], results of RT-PCR demonstrate that Nell2 in the retina is represented only by one splicing variant corresponding to the secreted form. EGF-like domain-containing proteins are mostly extracellular [21], but some are intracellular; for instance amphiregulin is localized to the nucleus [22] and prostaglandin endoperoxide H synthases to the endoplasmic reticulum and nuclear envelopes [23].

Nell2 is represented in the retinal extract by 90 kDa and 140 kDa proteins, which are proposed to be non-glycosylated and glycosylated Nell2, respectively. Both products were also present in cortex extract that was used in this experiment as a positive control. This data is in full agreement with an earlier report analyzing expression of Nell2 in various neuronal tissues including the olfactory bulb, hippocampus, cerebral cortex, thalamus, hypothalamus, cerebellum, and medulla [24]. The distribution of these proteins in the retina was similar to that observed in the olfactory bulb, hippocampus and cerebral cortex, where a 90 kDa product was more abundant than 140 kDa. The opposite pattern was present in the hypothalamus. Interestingly, the recombinant Nell2 expressed in cultured cells exist only as a 140 kDa glycosylated protein. Digestion with N-glyconase converts the 140 kDa product into 90 kDa protein [4]. With respect to the cellular specificity of Nell2 expression in the retina, the fact that in RGC-deficient (due to optic nerve axotomy) retinas the immunoreactive products corresponding to Nell2 were hardly detectable provides additional evidence that this protein is expressed in RGCs.

The dominant expression in early post-natal stages, as compared to the adult stage, suggests the role of Nell2 in neuron differentiation, including neurite extension and synapse formation [8], [24]. In the adult rat brain Nell2 expression was restricted to neurons and was not detected in glial cells and white matter, suggesting that Nell2 may also play an important role in differentiated neurons. Since the mechanism of function of Nell2 is unknown, we performed MS analysis to identify proteins interacting with Nell2. One protein, Macf1, was consistently present in IP reactions carried out with Nell2 antibodies and retinal lysate. Macf1 is a highly conserved ubiquitously expressed cytoskeletal linker protein containing N-terminal actin-binding domains, followed by plakin domains, extended spectrin repeat domains, EF-hand motifs, and C-terminal microtubule-binding domains [25]. High levels of Macf1 were detected in neuronal tissues [26]. Mice with standard MACF1 knockout (KO) are embryonic lethal by E10, whereas conditional KO animals die within 24–36 h after birth [27]. Multiple developmental brain defects, including a disorganization of the cerebral cortex, heterotopia of the hippocampal pyramidal layer, aplasia of the corpus callosum and anterior and hippocampal commissures, altered shapes of the lateral ventricles, and hypotrophy and disorganization of thalamocortical fibersin, were observed in mice with a conditional Macf1 KO [27]. The observed developmental defects were related to positive regulation of the Wnt signaling pathway: Macf1 associated with the core canonical Wnt protein complex containing Axin, Apc, Gsk3β, and β-catenin, and may be involved in the translocation of the proteins of the Axin complex from the cytoplasm to the plasma membrane [28]. Although not confirmed by experimental data, we can hypothesize that Macf1 assists Nell2 translocation to synaptic terminals and its possible function in axonal transport, release and the recycling of synaptic vesicles [29].

In addition to differentiation and proliferation of neuronal cells, Nell2 has been reported to promote cell survival [5], [16], [17], [18]. Aihara et al. reported that Nell2 promotes survival of primary cultured hippocampal and cortical neurons [5]. They found that treatment with Nell2 increased JNK phosphorylation and decreased phosphorylation of Erk, suggesting that Nell2-induced neuronal survival is partly due to modulation of mitogen-activated protein kinase (Mapk) activities. Nell2 has also been shown to be involved in promoting the neuronal survival required for the formation of a sexually dimorphic nucleus of the preoptic area in male rats [16]. Estrogen-induced survival of neuroprogenitor HiB5 cells may be mediated in part by trans-activation of Nell2 [17]. The blockade of Nell2 endogenous synthesis in HiB5 cells resulted in a decrease in Erk1/2 phosphorylation and in a loss of the cell survival effect. These data suggest that the Nell2 transcriptional activation by estrogen contributes to estrogen-mediating cell survival via the Erk signaling pathway.

Identification of new factors for RGC neuroprotection is of fundamental importance, since the degeneration of these cells and their axons in the optic nerve is the cause of vision loss in various optic neuropathies including glaucoma [30], [31], [32]. Since the exact molecular pathways of RGC death in glaucomatous neurodegeneration are not well understood, several avenues of RGC neuroprotection are being investigated, including blocking glutamate excitotoxicity, stabilizing Ca*^2+^* homeostasis, inhibiting nitric oxide production, supplying neurotrophins, enhancing the stress protein response, preventing apoptosis, improving blood flow to the optic nerve, and modulating immunologic status by vaccination [33], [34], [35], [36], [37], [38], [39]. Earlier, we have analyzed changes in gene expression associated with glaucomatous retinas in a rat model of a disease (unpublished data). Based on these data, we evaluated the role of several genes, including crystallins alphaA and alphaB and thiredoxins 1 and 2, the alterations in expression of which could affect RGC survival [40], [41], [42]. In this study, we tested our hypothesis that Nell2 may support RGC survival. The hypothesis was based on our findings indicating that Nell2 expression in the retina is restricted to RGCs and on earlier observations demonstrating a Nell2-mediated cell-protective effect. We used the rat optic nerve axotomy model, since it is characterized by highly reproducible, rapid and specific degeneration of RGCs. Our data indicate that Nell2 overexpression supports the survival of RGCs after axotomy. The cell-protective effect was more evident in the upper nasal retina where the ELP-mediated RGC transfection efficiency was approximately 49%. In this region, Nell2 transfection increase the number of survived RGCs by approximately 58% compared to non-transfected or EGFP-transfected contralateral axotomized retinas. We believe that the neuroprotective effect of Nell2 observed in this study could be even higher if the RGC transfection across the retina was more efficient. This pattern of regional ELP-mediated transfection was also observed earlier in our study on the role of thioredoxins in survival of RGCs. An important advantage of relatively efficient focal transfection and subsequent cell protection in the transfected region is that the other parts of the retina can serve as internal controls eliminating concerns related to possible variations in RGC density between animals and between two eyes of the same animal.

In summary, in the present study, we have demonstrated that Nell2 expression at both mRNA and protein levels is localized to RGCs in the retina. It is expressed as a cytosolic protein with a MW of approximately 140 kDa and 90 kDa corresponding to glycosylated and non-glycosylated proteins, respectively. The expression of Nell2 mRNA and protein in RGC-deficient retinas 2 weeks after optic nerve axotomy was dramatically decreased to almost non-existent levels, supporting the Nell2 mRNA and protein cellular localization data. The neuroprotective effect of Nell2 was clearly demonstrated in axotomized retinas, in which the overexpression of this gene in approximately 49% of transfected RGCs resulted in almost 58% increase in RGC survival compared to non-transfected or pEGFP-transfected controls.

## Materials and Methods

### ONT model

The use of animals for this study was approved by the Animal Research Committee of the University of California, Los Angeles and was performed in compliance with the Association for Research in Vision and Ophthalmology Statement for the Use of Animals in Ophthalmic and Vision Research. This study was specifically approved by the University of California, Los Angeles Animal Research Committee.

To generate the optic nerve transection (ONT) model, the animals (adult male Wistar rats weighing 250–300 g) were anesthetized by inhalation of an isofluroane (1.5–3.5%) in oxygen, the optic nerve was exposed through a lateral conjunctival incision, and the optic nerve sheath was incised 2 mm longitudinally, starting 3 mm behind the eye. A cross section of the optic nerve was made through the opening of the optic nerve sheath, with care not to damage the adjacent blood supply. The conjunctival incision was sutured and Tobrex ophthalmic ointment (tobramycin; Alcon, Fort Worth, TX) was applied topically. The ONT procedure was performed on one eye of each rat. Animals were euthanized two weeks after the procedure and eyeballs were enucleated and bisected. Retinas were dissected from the eyeballs for counting cells in the GCL, RNA isolation, ISH, immunoblot and IHC.

### RGC counting

RGCs were retrogradely labeled by placing a piece of Gelfoam soaked with 6% FG (Fluorochrome, Denver, CO) to the proximal cut surface of the optic nerve at the time of optic nerve axotomy. One day later, the eyes were enucleated and immersed in 4% paraformaldehyde in a 0.1 M phosphate buffer for 1 hour, the retinas were dissected, mounted on glass slides and divided into superotemporal, inferotemporal, superonasal, and inferonasal quadrant. Three sampling fields (0.32 mm×0.24 mm) were collected at each region 1, 2, and 3 mm from the center of the optic nerve in each retinal quadrant under fluorescent microscopy (LSM410, Carl Zeiss, Oberkochen, Germany) at 200× magnification. The numbers of RGCs in thirty-six sampling fields from each retina were counted and averaged. Morphologically distinguishable glial cells (bright and small cells) were not counted. Quantification of RGCs was carried out in a masked manner.

### *In situ* hybridization

The total retinal RNA and sequence-specific primers 5′- ACAGTTGACCTCTCCTGCTG (forward) and 5′-TCATTCCACGGCTTCAGTGAG (reverse) were used in RT-PCR to obtain a Nell2 DNA fragment. DNA fragment was subcloned into pCRII-Topo vector (Invitrogen, Carlsbad, CA), which contains sequences for Sp6 and T7 promoters. Template DNA was prepared by linearizing the plasmid DNA with the Bam HI or Xho I restriction enzymes. Digoxigenin (DIG)-labeled antisense and sense cRNA probes were synthesized by *in vitro* transcription with either T7 or Sp6 RNA polymerase according to the manufacturer's protocol (Roche Applied Science, Indianapolis, IN). Sense RNA probes were used in these experiments as the negative control.

ISHs were performed according to the standard protocol with some modifications. Briefly, 10 um-thick frozen sections fixed in 4% paraformaldehyde/phosphate-buffered saline (PBS) were washed with PBS for 30 min, and equilibrated for 15 min in 5× SSC (0.75 M NaCl, 0.075 M Na-Citrate). Prehybridization was carried out for 2 h in a solution containing 50% formamide, 5× SSC and 40 ug/ml salmon sperm DNA. Sections were then hybridized for 12–24 h in a humid chamber at 60°C in a prehybridization solution, with the addition of the DIG-labeled Nell2 cRNA probes at a concentration of 400 ng/ml. Following hybridization, slides were washed twice with 2× SSC and twice with 0.1× SSC at 65°C for 1 h. Immunological detection was performed with alkaline phosphatase (AP) conjugated anti-DIG antibodies at 4°C for 12–14 h. Following incubation with the DIG-AP antibody, color staining was developed by incubation sections with NBT/BCIP (Roche Applied Science).

### mRNA analysis

The total retinal RNA was extracted with RNAzol B (Tel-Test, Friendswood, TX), DNase treated with Turbo DNA-free (Ambion, Austin, TX) and purified with RNeasy MiniElute Cleanup kit (Qiagen, Valencia, CA). The 1^st^ strand cDNA from 5 micrograms of the total RNA was reverse transcribed with oligo-dT primer and M-MuLV (NEB, Beverly, MA). The cDNA was amplified by PCR with primers specific to the target sequence at the following conditions: 3 min at 95°C; 25 cycles of denaturing (95°C for 30 s), annealing (60°C for 15 s), and extension (72°C for 30 s); and a final extension of 7 min at 72°C. First strand cDNA dilutions in the PCR were adjusted to be within the linear range of amplification. To confirm that equivalent amounts of 1^st^ strand were included in the assay, β-actin was used as a standard. The following primers were used in semi-qPCR: for Nell2, 5′-ACAGTTGACCTCTCCTGCTG (forward) and 5′-TCATTCCACGGCTTCAGTGAG (reverse); and for β-actin, 5′-CTAGACTTCGAGCAAGAGATGGCCACT (forward) and 5′-TAGGAGCCAGGGCAGTAATCTCCTTCT (reverse).

To identify Nell2 splicing variants, 1^st^ strand retinal cDNA was used as a template in a PCR with 5′-ACTGAGACGATGCACGCCATG (forward) and 5′-TGGATCTGTTTCAGGGTCACC (reverse) Nell2-specific primers. PCR conditions following: 3 min at 95°C; 30 cycles of denaturing (95°C for 30 s), annealing (55°C for 30 s), and extension (72°C for 45 s); and a final extension of 10 min at 72°C.

### Immunoblot analysis

Aliquots of 2–10 ug of detergent-soluble retinal protein were separated on a 12% SDS-polyacrylamide gel and transferred to the membrane (Immobilon-P; Millipore, Billerica, MA). After incubation with primary antibodies to Nell2 (1∶200; Sigma, St Louis, MO or 1∶2000; Santa Cruz Biotechnology, Santa Cruz, CA,) or MACF1 (1∶2000, Santa Cruz Bioteachnology), the membranes were treated with peroxidase-conjugated secondary antibodies (Santa Cruz Biotechnology) for 1 hour and the immunoreactive bands were detected by chemiluminescence with ECL Western Blotting Detection Reagents (GE Healthcare Biosciences, Piscataway, NJ). To confirm equal protein loading of retinal lysates from different animals, membranes were reprobed with a monoclonal antibody to a housekeeping gene, GAPDH (1∶10000; Sigma).

### Immunohistochemistry

Five rats were used for IHC. Ten-micrometer thick retinal sections were incubated with primary mouse monoclonal anti-Nell2 antibody (provided by Dr. N. Saito) diluted 1∶500 overnight at 4°C. Sections were washed with 0.01 M PBS/0.2% Triton X-100 for 30 min and incubated with fluorescent Alexa Fluor 568 dye labeled goat anti-mouse IgG (Molecular Probes, Eugene, OR) diluted 1∶2000 for 1 hour at room temperature. Sections were mounted and the digital images were captured. The specificity of the immunoreaction was controlled by the exclusion of the primary antibody.

### Immunoprecipitation

Five rats were used for IP. Rat retinas and cortexes were homogenized in RIPA buffer (150 mM NaCl, 1% IGEPAL CA-630, 0.5% sodium deoxycholate, 0.1% SDS, 50 mM Tris, 1 mM PMSF) and the samples were centrifuged at 12000×g for 10 minutes at 4°C. IP was carried out with Immunoprecipitation Starter Pack (GE Healthcare), according to the manufacturer's instructions. Briefly, the supernatants were incubated with antibodies for Nell2 (Santa Cruz Biotechnology) or MACF1 (Santa Cruz Biotechnology) 1 h at 4°C, followed by incubation with Protein G Sepharose 4 Fast Flow for another 1 h at 4°C. After samples were washed three times with ice-cold wash buffer and the supernatants were removed by centrifugation at 12,000×g for 1 min, proteins were precipitated. The proteins were then isolated from the beads using immunoblot loading buffer for 4 min at 95°C, separated on a 7% SDS-polyacrylamide gel (Bio-Rad, Hercules, CA) and processed for Coomassie brilliant blue or Silver staining (Thermo Scientific, Rockford, IL), according to the manufacturer's instructions.

### Proteome analysis by matrix-assisted laser desorption/ionisation-time of flight mass spectrometry (MALDI-TOF MS)

Proteins were in-gel digested using trypsin (Roche Applied Science) according to manufacturer's protocol. After digestion, the gel pieces were extracted with 50% acetonitrile (ACN), 50% 0.1% formic acid (FA) (v/v) for 15 min. The supernatant was collected and the gel pieces were covered with 5% FA for 15 min before the same volume of ACN was added. After incubation for 10 min, the supernatant was collected. The pooled supernatants were then dried in a vacuum centrifuge and stored at −20°C. The trypsin-digested peptides concentrated by Ziptip C18 (Millipore) were placed on the anchor chip of a MALDI-TOF MS (Ultrafrex, Bruker Daltonics, Bremen, Germany) together with 100 fmol of a bradykinin fragment (m/z of 757; Sigma) as an internal control and 0.3 µg of 4-hydroxy-α-cinnamic acid matrix. Next, mass spectra of peptide peaks were detected using the automatic linear positive mode for simple comparison between the sample groups. The MS analysis was then performed using reflector mode to obtain accurate masses for the peptides. Finally, the MS/MS (TOF/TOF) analysis and subsequent sequence searching using Mascot were performed to identify the sequences of peptides of interest. A comparative analysis of the mass spectra of the peptide peaks was performed by using ClinProt Tools software v.1.0 (Bruker Daltonics). The intensities of the detected peptides were normalized using that of the bradykinin fragment.

### Nell2 expression construct

Enhanced green fluorescent protein (EGFP)-fused Nell2 expression construct was prepared in a pEGFP-N3 vector (Clontech, Mountain View, CA). The Nell2 coding region was obtained by RT-PCR with sequence-specific primers containing Eco RI and Bam HI restriction sites integrated into the forward (Nell2-1, 5′- CTAGAATTCGAGACGATGGACTGAGACGAT) and reverse (Nell2-2, 5′- ATATGGATCCCAGCTCCTGGAGGCAC) primers, respectively. First strand retinal cDNA synthesized with oligo-dT primer as described above was used as a template in PCR. The PCR product was digested with the Eco RI and Bam HI and cloned into pEGFP-N3 vector upstream of EGFP open reading frame.

### Electroporation and the efficiency of RGC transfection

pEGFP or pNell2-EGFP expression constructs were delivered to the retina by ELP as described previously with minor modifications [43], [44], [45]. Briefly, animals were anesthetized with an intramuscular injection of 0.8 ml/kg of a cocktail containing 5 ml ketamine (100 mg/ml), 2.5 ml xylazine (20 mg/ml), 1.0 ml acepromazine (10 mg/ml), and 1.5 ml normal saline. Twenty ug (4 ul) of plasmid DNA was injected under stereo-microscopy into the vitreous cavity 0.5 mm posterior to the limbus. Ten minutes later, the cathodal electrode was placed on the cornea, and an 18G-needle anodal electrode was inserted subcutaneously at the middle of the forehead. Two sets of 5 electric pulses with 10 min interval were generated with ECM 830 Pulse Generator (BTX, San Diego, CA) and delivered to the animals. Pulse parameters were as followed: electric field strength of 6 V/cm, pulse duration of 100 ms, and a stimulation pattern of five pulses at a frequency of one pulse/second.

The efficiency of RGC transfection was evaluated 7 days after ELP. RGCs were retrogradely labeled with FG, as described above, 5 days after ELP. FG and EGFP were visualized under fluorescence microscopy with a wide-band ultraviolet (UV) excitation filter (330–390 nm excitation and 420–480 nm emission) and with a green spectral filter (460–490 nm excitation and 510–550 nm emission), respectively. The efficiency of RGC transfection was determined by comparing the number of EGFP-positive cells colocalized with FG-labeled RGCs with the total number of FG-labeled RGCs at 1, 2 and 3 mm from the center of the optic nerve in each of the retinal quadrants (see RGC Counting above).

### Statistical Analysis

Data are presented as the mean ± standard deviation (SD). Differences among groups were analyzed by one-way ANOVA, followed by the Scheffé or Mann-Whitney test. P<0.05 was considered statistically significant.
